# Supplementary material for: A novel decoy strategy for polymyxin resistance in Acinetobacter baumannii
Source: eLife. 2021 Jun 28;10:e66988. doi: 10.7554/eLife.66988 (PMC8324293; doi:10.7554/eLife.66988)

**Figure 3-source data 2**. Images of selected spots in proteomics analysis (Lab-WT, Lab-WT+1/2 MIC, PMR^High^ and PMR^High^ +1/2MIC)


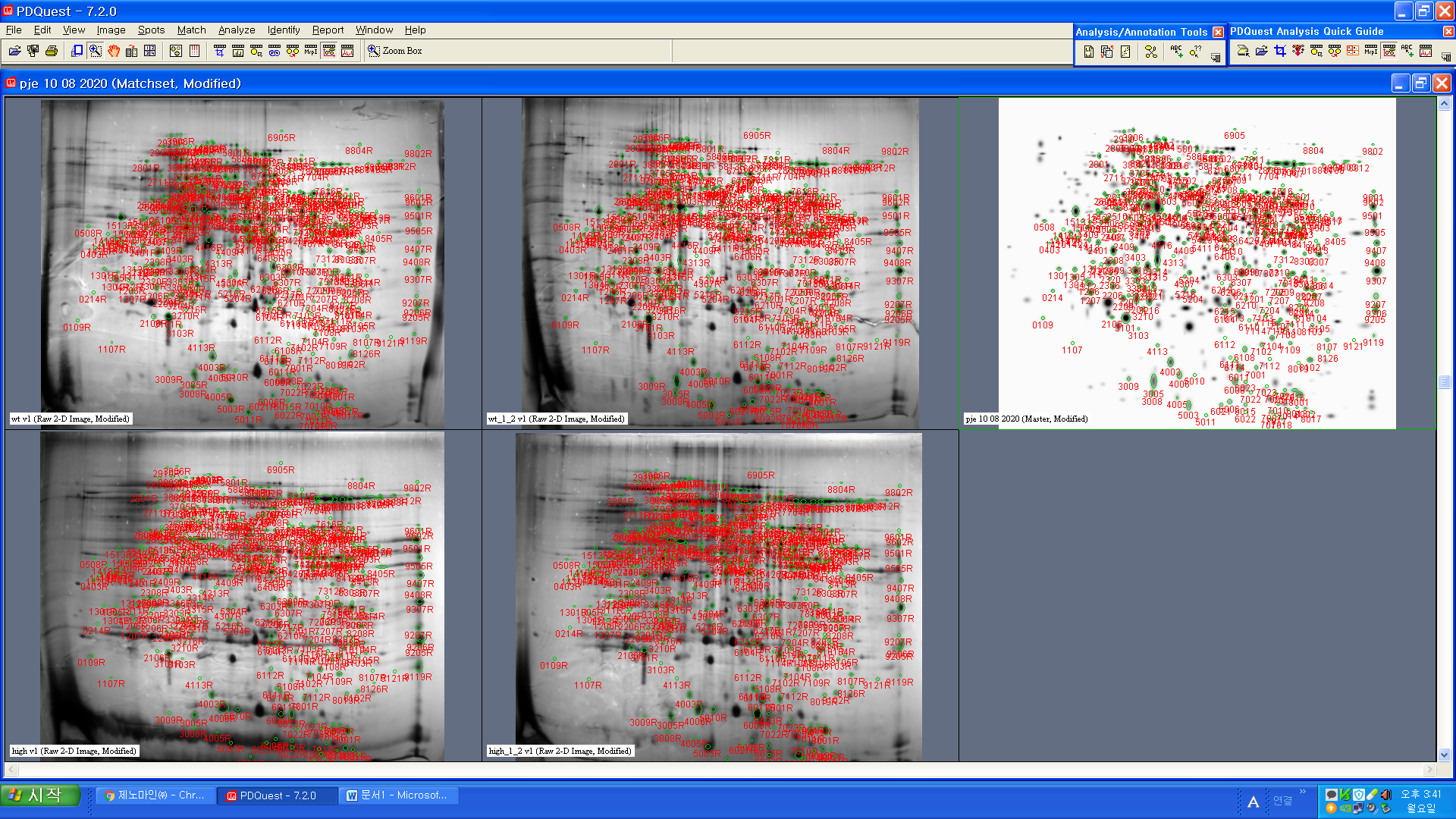

Supplement: Figure 3—source data 2. [file elife-66988-fig3-data2.docx]
