# Supplementary material for: A novel decoy strategy for polymyxin resistance in Acinetobacter baumannii
Source: eLife. 2021 Jun 28;10:e66988. doi: 10.7554/eLife.66988 (PMC8324293; doi:10.7554/eLife.66988)
Supplement: Supplementary file 1. — (a) Findings from sequence analysis of mutated genes in the genomes of PMRLow and PMRHigh isolates. Mutations were identified using the MUMmer program. Additional PCR reactions using Pfu DNA polymerase were performed to confirm mutational positions. All position numbers are based on the genes of A. baumannii ATCC 17978. (b) Whole-genome sequence analyses of Lab-WT, PMRLow, and PMRHigh isolates using the PacBio sequencing technique. The genome sequences were downloaded from EzGenome and then their chromosomes were analyzed using the CLgenomics program. (c) Lists of deleted regions in the chromosomes of PMRLow and PMRHigh. The genome sequences were compared to the reference genome, A. baumannii ATCC 17978, and whole genomes were aligned using Snapgene. (d) Identification of antibiotic-resistance genes (ARGs) in Lab-WT, PMRLow, and PMRHigh strains. Totally 20 ARGs were detected using the CLC Genomics Workbench v.10.0.1 (QIAGEN, Germany). (e) Identification of insertion sequence (IS) elements in the Lab-WT, PMRLow, and PMRHigh. All parameters were set as default. IS elements were analyzed using the IS finder (https://isfinder.biotoul.fr/blast.php). (f) All information on the acquisition of RNA-seq data in this study. Total RNAs were extracted from exponentially grown Lab-WT, Lab-WT strain + polymyxin B (PMB), PMRHigh and PMRHigh strain + PMB. RPKM represents reads per kilobase of transcript per million mapped sequence reads. (g) List of genes that were up- or downregulated in PMRHigh, Lab-WT + PMB, and PMRHigh + PMB in comparison with control strains. Up- (fold change > 1.4, RPKM > 50) or downregulated (fold change < 0.7, RPKM > 50) genes were indicated. The intersection area and numbers are described in Figure 3—figure supplement 1. All locus tags are based on the genes of A. baumannii ATCC 17978. *TM = transmembrane region, ND = not detected. (h) Relative abundance of proteins in three conditions that increased outer membrane vesicles (OMVs): Lab-WT + PMB, PMRHigh + P [file elife-66988-supp1.docx]

**A novel decoy strategy for polymyxin resistance in *Acinetobacter baumannii***

Jaeeun Park^a^, Misung Kim^a^, Bora Shin^b^, Mingyeong Kang^a^, Jihye Yang^a^, Tae Kwon Lee^c^ and Woojun Park^a^*

^a^Laboratory of Molecular Environmental Microbiology, Department of Environmental Science and Ecological Engineering, Korea University, Seoul 02841, Republic of Korea

^b^Department of Microbiology and Immunology, School of Medicine, National University of Singapore, Singapore

^c^Department of Environmental Engineering, Yonsei University, Wonju 26493, Korea

**Running title**: OMV production in *Acinetobacter baumannii*

**Keywords**: *Acinetobacter baumannii*; LPS modifications; outer membrane vesicles; two-component regulatory systems; experimental evolution; polymyxin-resistance

***Corresponding author:** Dr. Woojun Park, Department of Environmental Science and Ecological Engineering, Korea University, Seoul 02841, Republic of Korea

**E-mail:** wpark@korea.ac.kr

**Fax:** +82-2-953-0737

**Phone:** +82-2-3290-3067

**Supplementary File 1a.**

| **Product name** | **Nucleotide change** | **Amino acid change** |
| --- | --- | --- |
| **Mutation in PMR^Low^ and PMR^High^** |  |  |
| Two-component sensor histidine kinase (*pmrB*) | A1058T | N353I (PMR^Low^) |
|  | C704T | T235I (PMR^High^) |
| **Mutation in PMR^Low^** |  |  |
| GGDEF domain-containing protein (*dgcB*) | ΔA1707 | Internal stop codon |
| DNA translocase (*ftsK*) | ΔG2631 | Internal stop codon |
| **Mutation in PMR^High^** |  |  |
| 5′/3′-nucleotidase (*surE*) | ΔA451 | Internal stop codon |
| Ribosomal-protein-alanine *N*-acetyltransferase (*rimJ*) | ΔA392 | Internal stop codon |
| ABC transporter substrate-binding protein (*mlaC*) | ΔT95 | Internal stop codon |
| Fimbrial biogenesis protein (*fimT*) | ΔA365 | Internal stop codon |
| Cell division protein (*ftsL*) | ΔA47 | Internal stop codon |
| peptidase M23/Uracil-DNA glycosylase (*udg*) | Δ576-580 | Internal stop codon |
| Beta-ketoacyl-[acyl-carrier-protein] synthase I (*fabH*) | ΔC305 | Internal stop codon |
| Site-specific tyrosine recombinase (*xerD*) | ΔT158 | Internal stop codon |
| Ferrous iron transporter B (*feoB*) | ΔG435 | Internal stop codon |
| GTP pyrophosphokinase (*relA*) | ΔC239 | A80V |
| Citrate-proton symporter | ΔT35 | Internal stop codon |
| 16S rRNA (cytidine(1402)-2'-O)-methyltransferase (*rsmI*) | ΔT590 | Internal stop codon |
| Dihydrofolate reductase (*folA*) | ΔT258 | Internal stop codon |
| Glutamine aminotransferase (*guaA*) | ΔT87 | Internal stop codon |

**Supplementary File 1b.**

| **Strain** | **CDS** | **Sequencing  depth of  coverage** | **Mean  CDS length (bp)** | **Median CDS length (bp)** | **GC ratio (%)** | **Chromosomal DNA (bp)** | **Mean length of intergenic  region (bp)** | **N50 (bp)** | **No. of  rRNA  genes** | **No. of  tRNA  genes** |
| --- | --- | --- | --- | --- | --- | --- | --- | --- | --- | --- |
| **Lab-WT (ATCC 17978)** | 3803 | 516 | 924.2 | 790 | 38.9 | 3,976,747 | 112.2 | 3,902,113 | 15 | 70 |
| **PMR^Low^** | 3678 | 299.8 | 947.5 | 816 | 38.9 | 3,971,618 | 132.8 | 3,971,618 | 18 | 72 |
| **PMR^High^** | 3762 | 247.19 | 930.2 | 804 | 38.9 | 3,955,017 | 138.5 | 3,955,017 | 18 | 72 |

**Supplementary File 1c.**

| Strain | Positions in  chromosome | Locus tag | Product |
| --- | --- | --- | --- |
| PMR^Low^ | Region 1  (680-700 kbp) | A1S_3508 | Hypothetical protein |
|  |  | A1S_3510 | Hypothetical protein |
|  |  | A1S_3514 | Hypothetical protein |
|  |  | A1S_3515 | Hypothetical protein |
|  |  | A1S_0629 | Hypothetical protein |
| PMR^Low^ | Region 2  (1,830-1,863 kbp) | A1S_3683 | Hypothetical protein |
|  |  | A1S_1582 | Transcriptional regulator Cro/CI family |
|  |  | A1S_1583 | Hypothetical protein |
|  |  | A1S_1584 | Hypothetical protein |
|  |  | A1S_3698 | Hypothetical protein |
|  |  | A1S_1597 | Phage tail tape measure protein lambda |
| PMR^High^ | Region 1  (680-700 kbp) | A1S_0626 | Hypothetical protein |
|  |  | A1S_3508 | Hypothetical protein |
|  |  | A1S_3509 | Hypothetical protein |
|  |  | A1S_3510 | Hypothetical protein |
|  |  | A1S_3514 | Hypothetical protein |
|  |  | A1S_3515 | Hypothetical protein |
|  |  | A1S_3517 | Hypothetical protein |
|  |  | A1S_3518 | Hypothetical protein |
|  |  | A1S_0629 | Hypothetical protein |
|  |  | A1S_3519 | Hypothetical protein |
|  |  | A1S_3520 | Hypothetical protein |
| PMR^High^ |  | A1S_3683 | Hypothetical protein |
|  |  | A1S_1581 | Methyltransferase putative |
|  |  | A1S_3685 | Hypothetical protein |
|  |  | A1S_3686 | Hypothetical protein |
|  |  | A1S_3687 | Hypothetical protein |
|  |  | A1S_1582 | Transcriptional regulator Cro/CI family |
|  |  | A1S_1583 | Hypothetical protein |
|  |  | A1S_1584 | Hypothetical protein |
|  |  | A1S_1585 | Putative replicative DNA helicase |
|  |  | A1S_3688 | Hypothetical protein |
|  |  | A1S_3689 | Hypothetical protein |
|  |  | A1S_3690 | Hypothetical protein |
|  |  | A1S_3691 | Hypothetical protein |
|  |  | A1S_3692 | Hypothetical protein |
|  |  | A1S_3693 | Hypothetical protein |
|  |  | A1S_3694 | Hypothetical protein |
|  | Region 2  (1,830-1,863 kbp) | A1S_3695 | Hypothetical protein |
|  |  | A1S_3696 | Hypothetical protein |
|  |  | A1S_1586 | Ethanol-stimulated virulence protein, EsvK1 |
|  |  | A1S_3697 | Hypothetical protein |
|  |  | A1S_3698 | Hypothetical protein |
|  |  | A1S_1587 | Ethanol-stimulated virulence protein, EsvK2 |
|  |  | A1S_1588 | Phage terminase-like protein large subunit |
|  |  | A1S_1589 | Hypothetical protein |
|  |  | A1S_1590 | Peptidase U35 phage prohead HK97 |
|  |  | A1S_1591 | Phage major capsid protein HK97 |
|  |  | A1S_3699 | Hypothetical protein |
|  |  | A1S_1592 | Putative Phage head-tail adaptor |
|  |  | A1S_1593 | Hypothetical protein |
|  |  | A1S_1594 | Hypothetical protein |
|  |  | A1S_1595 | Hypothetical protein |
|  |  | A1S_3700 | Hypothetical protein |
|  |  | A1S_3701 | Hypothetical protein |
|  |  | A1S_1596 | Hypothetical protein |
|  |  | A1S_1597 | Phage tail tape meausure protein lambda |
|  |  | A1S_3702 | Hypothetical protein |
|  |  | A1S_1598 | Hypothetical protein |
|  |  | A1S_1599 | Hypothetical protein |
|  |  | A1S_3703 | Hypothetical protein |
|  |  | A1S_3704 | Hypothetical protein |

**Supplementary File 1d.**

| **Gene** | **Identity (%)** | **Length** | **Position in chromosome** | **Predicted phenotype** |
| --- | --- | --- | --- | --- |
| *ADC-79* | 100 | 1152/1152 | 154172-155323 | Determinant of β-lactam resistance |
|  | 100 | 1152/1152 | 2619269-2620420 |  |
| *OXA-259* | 100 | 825/825 | 1643403-1644227 | Determinant of β-lactam resistance |
|  | 100 | 825/825 | 3168483-3169307 |  |
| *sul2* | 100 | 816/816 | 1272808-1273623 | Antibiotic target replacement protein |
|  | 100 | 816/816 | 3736874-3737689 |  |
| *amvA* | 97.50 | 1479 / 1479 | 3766657..3768135 | Efflux pump complex or subunit conferring antibiotic resistance |
|  | 97.50 | 1479 / 1479 | 2276735..2278213 |  |
| *abaQ* | 98.77 | 1305 / 1305 | 3507191..3508495 | Efflux pump complex or subunit conferring antibiotic resistance |
|  | 98.77 | 1305 / 1305 | 2017267..2018571 |  |
| *abaF* | 100 | 1290 / 1290 | 2959632..2960921 | Efflux pump complex or subunit conferring antibiotic resistance |
|  | 100 | 1290 / 1290 | 1434550..1435839 |  |
| *adeL* | 99.31 | 1014 / 1014 | 80072..81085 | Protein(s) and two-component regulatory system modulating antibiotic efflux |
|  | 99.31 | 1014 / 1014 | 2545169..2546182 |  |
| *adeN* | 99.69 | 654 / 654 | 3661056..3661709 | Protein(s) and two-component regulatory system modulating antibiotic efflux |
|  | 99.69 | 654 / 654 | 2171133..2171786 |  |
| *adeK* | 99.31 | 1455 / 1455 | 591688..593142 | Efflux pump complex or subunit conferring antibiotic resistance |
|  | 99.31 | 1455 / 1455 | 3056790..3058244 |  |
| *adeI* | 99.60 | 1251 / 1251 | 587249..588499 | Efflux pump complex or subunit conferring antibiotic resistance |
| *abeM* | 99.18 | 1347 / 1347 | 433763..435109 | Efflux pump complex or subunit conferring antibiotic resistance |
|  | 99.18 | 1347 / 1347 | 1958865..1960211 |  |
| *adeA* | 97.98 | 1191 / 1191 | 3407427..3408617 | Efflux pump complex or subunit conferring antibiotic resistance |
|  | 97.98 | 1191 / 1191 | 1917502..1918692 |  |
| *adeG* | 97.38 | 1221 / 1221 | 81301..82518 | Efflux pump complex or subunit conferring antibiotic resistance |
|  | 97.38 | 1221 / 1221 | 2546398..2547615 |  |
| *adeB* | 98.10 | 3108 / 3108 | 3404320..3407427 | Efflux pump complex or subunit conferring antibiotic resistance |
|  | 98.10 | 3108 / 3108 | 1914395..1917502 |  |
| *adeJ* | 99.37 | 3177 / 3177 | 588512..591688 | Efflux pump complex or subunit conferring antibiotic resistance |
|  | 99.37 | 3177 / 3177 | 3053614..3056790 |  |
| *abeS* | 88.79 | 330 / 330 | 76433..76762 | Efflux pump complex or subunit conferring antibiotic resistance |
|  | 88.79 | 330 / 330 | 2541530..2541859 |  |
| *adeH* | 99.24 | 1452 / 1452 | 85714..87165 | Efflux pump complex or subunit conferring antibiotic resistance |
|  | 99.24 | 1452 / 1452 | 2550811..2552262 |  |
| *adeR* | 98.79 | 744 / 744 | 3408763..3409506 | Protein(s) and two-component regulatory system modulating antibiotic efflux |
|  | 98.79 | 744 / 744 | 1918838..1919581 |  |
| *adeS* | 96.78 | 1086 / 1086 | 3409538..3410623 | Protein(s) and two-component regulatory system modulating antibiotic efflux |
|  | 96.78 | 1086 / 1086 | 1919613..1920698 |  |
| *adeF* | 97.30 | 3180 / 3180 | 82525..85704 | Efflux pump complex or subunit conferring antibiotic resistance |
|  | 97.30 | 3180 / 3180 | 2547622..2550801 |  |

**Supplementary File 1e**.

| Chromosomal DNA of the Lab-WT, PMR^Low^ and PMR^High^ | | | | |
| --- | --- | --- | --- | --- |
| Name | IS Family | Group | Origin | E-value |
| IS*Aba18* | IS3 | IS51 | *Acinetobacter baumannii* | 0 |
| IS*Aba1* | IS4 | IS10 | *Acinetobacter baumannii* | 0 |
| IS*Aba11* | IS701 | ISAba11 | *Acinetobacter baumannii* | 0 |
| IS*Aba12* | IS5 | IS903 | *Acinetobacter baumannii* | 0 |
| IS*Aba53* | IS5 | IS903 | *Acinetobacter baumannii* | 0 |
| IS17 | IS5 | IS903 | *Acinetobacter haemolyticus* | 0 |
| IS*Alw23* | IS3 | IS51 | *Acinetobacter lwoffii* | 0 |
| IS*Alw4* | IS3 | IS51 | *Acinetobacter lwoffii* | 0 |
| IS*Aba29* | IS3 | IS51 | *Acinetobacter baumannii* | 0 |
| IS*Alw5* | IS3 | IS51 | *Acinetobacter lwoffii* | 0 |
| IS*Aba19* | IS3 | IS51 | *Acinetobacter baumannii* | 0 |
| IS*Aba2* | IS3 | IS51 | *Acinetobacter baumannii* | 0 |
| IS*Aba33* | IS4 | IS10 | *Acinetobacter baumannii* | 0 |
| IS*Aba34* | IS3 | IS51 | *Acinetobacter baumannii* | 0 |
| IS*Acsp3* | IS3 | IS3 | *Acinetobacter* sp. | 0 |
| IS*Aba59* | IS5 | IS903 | *Acinetobacter baumannii* | 0 |
| IS*1236* | IS3 | IS3 | *Acinetobacter calcoaceticus* | 0 |
| IS*Alw26* | IS3 | IS3 | *Acinetobacter lwoffii* | 0 |
| IS*Aba13* | IS5 | IS903 | *Acinetobacter baumannii* | 0 |
| IS*Aba5* | IS5 | IS903 | *Acinetobacter baumannii* | 0 |
| IS*Aha1* | IS5 | IS903 | *Acinetobacter haemolyticus* | 0 |
| IS*Alw1* | IS5 | IS903 | *Acinetobacter lwoffii* | 0 |
| IS*Aba40* | IS5 | IS903 | *Acinetobacter baumannii* | 1.00E-164 |
| IS*Aha3* | IS5 | IS903 | *Acinetobacter haemolyticus* | 5.00E-155 |
| IS*Aha2* | IS5 | IS903 | *Acinetobacter haemolyticus* | 8.00E-154 |
| IS*Ajo1* | IS5 | IS903 | *Acinetobacter johnsonii* | 7.00E-145 |
| IS*Alw32* | IS5 | ISL2 | *Acinetobacter lwoffii* | 3.00E-141 |
| IS*Aba7* | IS5 | IS903 | *Acinetobacter baumannii* | 5.00E-124 |
| IS*Alw14* | IS5 | ISL2 | *Acinetobacter lwoffii* | 4.00E-103 |
| IS*Abe12* | IS3 | IS3 | *Acinetobacter bereziniae* | 1.00E-13 |
| IS*1301* | IS5 | IS427 | *Neisseria meningitidis* | 3.00E-11 |
| IS*Ppu33* | IS3 | IS51 | *Pseudomonas putida* | 5.00E-04 |
| IS*Stma17* | IS3 | IS51 | *Stenotrophomonas maltophilia* | 5.00E-04 |
| IS*Mca3* | IS3 | IS51 | *Methylococcus capsulatus* | 5.00E-04 |
| IS*1416* | IS3 | IS51 | *Burkholderia glumae* | 5.00E-04 |
| IS*Ate1* | IS5 | IS903 | *Acidovorax temperans* | 0.002 |
| IS*Ali4* | IS3 | IS51 | *Azospirillum lipoferum* | 0.002 |
| IS*Rme15* | IS3 | IS51 | *Ralstonia metallidurans* | 0.002 |
| IS*Pmi1* | IS3 | IS51 | *Proteus mirabilis* | 0.007 |
| IS51 | IS3 | IS51 | *Pseudomonas syringae* | 0.007 |
| IS*Cce4* | IS3 | IS3 | *Clostridium cellulolyticum* | 0.028 |
| IS*Rtr2* | IS3 | IS51 | *Rhizobium tropici* | 0.028 |
| IS*Aac2* | IS5 | IS427 | *Actinobacillus actinomycetemcomitans* | 0.028 |
| IS*Bt3* | IS3 | IS51 | *Burkholderia thailandensis* | 0.11 |
| IS*Cpi1* | IS5 | IS903 | *Chromobacterium piscinae* | 0.11 |
| IS*Afe7* | IS3 | IS51 | *Acidithiobacillus ferrooxidans* | 0.11 |
| IS*Jsp1* | IS3 | IS51 | *Janthinobacterium* sp. | 0.11 |
| IS*Pye52* | IS3 | IS51 | *Paracoccus yeei* | 0.44 |
| IS*Pre1* | IS5 | IS5 | *Pseudomonas resinovorans* | 0.44 |
| IS*Teha5* | IS4 | IS4Sa | *Tetragenococcus halophilus* | 1.7 |
| IS*Nov2* | IS5 | IS903 | *Novosphingobium* sp. | 1.7 |
| IS*Mex33* | IS3 | IS51 | *Methylobacterium extorquens* | 1.7 |
| IS*Osp1* | IS4 | IS4Sa | *Oceanobacter* sp. | 1.7 |
| IS*Azsp1* | IS3 | IS51 | *Azotobacter* sp. | 1.7 |
| IS*Pst4* | IS3 | IS51 | *Pseudomonas stutzeri* | 1.7 |
| IS*Alw15* | IS5 | ISL2 | *Acinetobacter lwoffii* | 6.9 |
| IS*Pye26* | IS110 | IS1111 | *Paracoccus yeei* | 6.9 |
| IS*Bth21* | IS5 | IS427 | *Bacillus thuringiensis* | 6.9 |
| IS*Hce1* | IS1595 | ISPna2 | *Helicobacter cetorum* | 6.9 |
| IS*Aur1* | IS1595 | ISPna2 | *Actinobacillus ureae* | 6.9 |
| IS*Cig1* | IS1595 | ISPna2 | *Campylobacter iguaniorum* | 6.9 |
| IS*Aisp8* | IS1595 | ISSod11 | *Acidovorax* sp. | 6.9 |
| IS*Bvi8* | IS5 | IS903 | *Burkholderia vietnamiensis* | 6.9 |
| IS*Asp20* | IS200/IS605 | IS200 | *Anabaena* sp*.* | 6.9 |
| IS*Hli3* | IS200/IS605 | IS1341 | *Halohasta litchfieldiae* | 6.9 |
| IS*Sri1* | ISKra4 | ISKra4 | *Streptomyces rimosus* | 6.9 |
| IS*Cap2* | IS5 | IS903 | *Candidatus Accumulibacter* | 6.9 |
| IS*Wosp5* | IS4 | IS231 | *Wolbachia* sp*.* | 6.9 |
| IS*Spu20* | IS5 | IS903 | *Shewanella putrefaciens* | 6.9 |
| IS*Cpe8* | IS1595 | ISPna2 | *Clostridium perfringens* | 6.9 |
| IS*Acma22* | IS701 | ISAba11 | *Acaryochloris marina* | 6.9 |
| IS*Cba1* | IS1595 | ISPna2 | *Clostridium bartlettii* | 6.9 |
| IS*Kpn4* | IS110 | IS1111 | *Klebsiella pneumoniae* | 6.9 |
| IS*Efa8* | IS3 | IS150 | *Enterococcus faecium* | 6.9 |
| IS*Afe1* | IS110 | IS1111 | *Acidithiobacillus ferroxidans* | 6.9 |
| IS*Sod2* | IS3 | IS407 | *Shewanella oneidensis* | 6.9 |
| IS*Brsp1* | IS3 | IS407 | *Bradyrhizobium* sp. | 6.9 |
| IS1302 | IS3 | IS150 | *Wolinella succinogenes* | 6.9 |
| pAB1 plasmid in Lab-WT | | | | |
| Name | IS Family | Group | Origin | E-value |
| IS*Alw13* | IS5 | IS427 | *Acinetobacter lwoffii* | 4e-50 |

**Supplementary File 1f**.

|  | Lab-WT | Lab-WT + PMB | PMR^High^ | PMR^High^ + PMB |
| --- | --- | --- | --- | --- |
| Total reads | 1,387,480 | 1,401,287 | 1,406,593 | 1,418,191 |
| Total mapped reads (%) | 97.6 | 97.7 | 98.6 | 97.8 |
| Average RPKM | 361.6 | 383.4 | 366.5 | 369.6 |
| Median RPKM | 338.8 | 317.2 | 326.0 | 311.1 |

**Supplementary File 1g.**

| **Regulation** | **Venn diagram Part** | **Locus tag** | **Gene** | **Site** | **TM** | **Products** |
| --- | --- | --- | --- | --- | --- | --- |
| Up | 1 | ATCC1_00077 | *DnaK* | Cytosol | ND | Chaperone protein DnaK |
| Up | 1 | ATCC1_00122 | *fkpA* | periplasm | ND | Peptidylprolyl isomerase |
| Up | 1 | ATCC1_00127 | *wbpB* | Cytosol | ND | UDP-*N*-acetyl-2-amino-2-deoxyglucuronate dehydrogenase |
| Up | 1 | ATCC1_00225 | *atpH* | Cytosol | ND | ATP synthase subunit delta |
| Up | 1 | ATCC1_00632 | *rplW* | Cytosol | ND | 50S ribosomal protein L23 |
| Up | 1 | ATCC1_00637 | *rplP* | Cytosol | ND | 50S ribosomal protein L16 |
| Up | 1 | ATCC1_00649 | *rplO* | Cytosol | ND | 50S ribosomal protein L15 |
| Up | 1 | ATCC1_00963 | *pmrC* | Inner membrane | TM | Lipid A phosphoethanolamine transferase |
| Up | 1 | ATCC1_00964 | *pmrA* | Cytosol | ND | Transcriptional regulatory protein PmrA |
| Up | 1 | ATCC1_01052 | *groL* | Cytosol | ND | 60 kDa chaperonin |
| Up | 1 | ATCC1_01563 | *cyoE* | Inner membrane | TM | Protoheme IX farnesyltransferase |
| Up | 1 | ATCC1_01578 | *yiaD* | Outer membrane | TM | Glycine-zipper family protein |
| Up | 1 | ATCC1_01620 | *miaA* | Cytosol | ND | tRNA dimethylallyltransferase |
| Up | 1 | ATCC1_01632 | *aldB* | Cytosol |  | Long-chain-aldehyde dehydrogenase |
| Up | 1 | ATCC1_01792 | *tusD* | Cytosol | ND | Sulfurtransferase TusD |
| Up | 1 | ATCC1_01850 | *grxD* | Cytosol | ND | Glutaredoxin |
| Up | 1 | ATCC1_01865 | *HyP* |  |  | Hypothetical protein |
| Up | 1 | ATCC1_02021 | *HyP* |  |  | Hypothetical protein |
| Up | 1 | ATCC1_02064 | *bdh* | Cytosol | ND | 3-hydroxybutyrate dehydrogenase |
| Up | 1 | ATCC1_02214 | *exbD1* | Inner membrane | TM | Biopolymer transport protein exbD1 |
| Up | 1 | ATCC1_02290 | *HyP* |  |  | Hypothetical protein |
| Up | 1 | ATCC1_02340 | *ansB* | Cytosol | ND | Glutamin-(asparagin-)ase |
| Up | 1 | ATCC1_02512 | *hcp1* | Extracellular |  | Protein hcp1 |
| Up | 1 | ATCC1_02642 | *loiP* | Cytosol | ND | Belongs to the peptidase M48 family |
| Up | 1 | ATCC1_02918 | *pgaB* | periplasm | ND | Poly-beta-1,6-*N*-acetyl-d-glucosamine N-deacetylase |
| Up | 1 | ATCC1_03012 | *coaD* | Cytosol | ND | Pantetheine-phosphate adenylyltransferase |
| Up | 1 | ATCC1_03514 | *rpoC* | Cytosol | ND | DNA-directed RNA polymerase |
| Up | 1 | ATCC1_03719 | *pgpA* | Outer membrane | TM | Phosphatidylglycerophosphatase |
| Up | 1 | ATCC1_03835 | *Hyp* |  |  | Hypothetical protein |
| Down | 1 | ATCC1_00610 | *mlaC* | Periplasm | ND | Intermembrane phospholipid transport system binding protein MlaC |
| Down | 1 | ATCC1_00872 | *ompA* | Outer membrane | TM | Outer membrane protein Omp38 |
| Down | 1 | ATCC1_01297 | *lpp* | Periplasm | ND | Lipoprotein NlpD/LppB like protein |
| Down | 1 | ATCC1_01335 | *dhbF* | Cytosol | ND | Dimodular nonribosomal peptide synthase |
| Down | 1 | ATCC1_01342 | *fatA* | Outer membrane | TM | Ferric anguibactin receptor |
| Down | 1 | ATCC1_01344 | *mbtB* | Cytosol | ND | Phenyloxazoline synthase MbtB |
| Down | 1 | ATCC1_01348 | *HyP* |  |  | Hypothetical protein |
| Down | 1 | ATCC1_02159 | *rhbD* | Cytosol | ND | Rhizobactin siderophore biosynthesis protein RhbD |
| Down | 1 | ATCC1_02622 | *ahpF* | Cytosol | ND | Alkyl hydroperoxide reductase subunit |
| Down | 1 | ATCC1_02964 | *bamE* | Outer membrane | TM | Outer membrane protein assembly factor BamE |
| Down | 1 | ATCC1_02991 | *fusA* | Outer membrane | TM | Elongation factor |
| Down | 1 | ATCC1_03271 | *cusC* | Outer membrane | TM | Cation efflux system protein CusC |

**Supplementary File 1h.**

| **Regulation** | **Venn diagram Part** | **Sample** | **Products** | **Gene** | **MASS (Kda)** | | **Expect** | | **Coverage** |
| --- | --- | --- | --- | --- | --- | --- | --- | --- | --- |
| Up | 1 | 7411 | Phosphoethanolamine transferase EptA domain protein, partial | *pmrC* | 14.784 | | 7.60E-08 | | 74.8% |
| Up | 1 | 7707 | Poly-beta-1,6-N-acetyl-D-glucosamine N-deacetylase PgaB | *pgaB* | 75.711 | | 5.01E-09 | | 29.80% |
| Up | 1 | 7808 | Poly-beta-1,6-N-acetyl-D-glucosamine N-deacetylase PgaB | *pgaB* | 73.429 | | 1.29E-08 | | 30.70% |
| Up | 1 | 7813 | Poly-beta-1,6-N-acetyl-D-glucosamine N-deacetylase PgaB | *pgaB* | 73.429 | | 6.29E-08 | | 23.50% |
| Up | 1 | 8701 | Serine-type D-Ala-D-Ala carboxypeptidase | *dacC* | 41.742 | | 3.10E-12 | | 11% |
| Up | 1 | 9407 | Alanine racemase | *alr* | 44.972 | | 2.20E-13 | | 45.80% |
| Up | 1 | 9501 | Alanine racemase | *alr* | 47.285 | | 5.04E-13 | | 45.80% |
| Up | 2 | 2506 | Alkyl hydroperoxide reductase C22 subunit | *ahpC* | 18.214 | | 1.60E-10 | | 53% |
| Up | 2 | 3502 | ND | ND | ND | | ND | | ND |
| Up | 2 | 5010 | 50S ribosomal protein L9 | *rplI* | 15.771 | | 1.60E-10 | | 64% |
| Up | 2 | 6006 | ND | ND | ND | | ND | | ND |
| Up | 2 | 6008 | 50S ribosomal protein L9 | *rplI* | 15.771 | | 4.00E-15 | | 68% |
| Up | 2 | 6011 | ND | ND | ND | | ND | | ND |
| Up | 2 | 6021 | ND | ND | ND | | ND | | ND |
| Up | 2 | 7517 | Citrate synthase | [gltA](https://www.uniprot.org/uniprot/B0VSK2) | 47.658 | 2.00E-11 | | 25% | |
| Up | 2 | 8405 | ND | ND | ND | ND | | ND | |
| Up | 2 | 9119 | Chorismate mutase | *pheA* | 19.38 | 3.10E-11 | | 29% | |
| Up | 2 | 9205 | ND | ND | ND | ND | | ND | |
| Up | 3 | 2505 | ND | ND | ND | ND | | ND | |
| Up | 3 | 7410 | N-acetyl-gamma-glutamyl-phosphate reductase | *argC* | 38.212 | 1.60E-08 | | 36% | |
| Up | 3 | 8001 | ND | ND | ND | ND | | ND | |
| Up | 4 | 2711 | ND | ND | ND | ND | | ND | |
| Up | 4 | 4006 | Nucleoside diphosphate kinase | *ndk* | 15,462 | 2.60E-05 | | 49% | |
| Up | 4 | 6104 | ND | ND | ND | ND | | ND | |
| Up | 4 | 6113 | ND | ND | ND | ND | | ND | |
| Up | 4 | 7007 | ND | ND | ND | ND | | ND | |
| Up | 4 | 7106 | Uncharacterized protein | ND | 38.13 | ND | | 23% | |
| Up | 4 | 7418 | ND | ND | ND | ND | | ND | |
| Up | 4 | 7706 | ND | ND | ND | ND | | ND | |
| Up | 4 | 7806 | ND | ND | ND | ND | | ND | |
| Up | 4 | 9206 | ND | ND | ND | ND | | ND | |
| Down | 1 | 2512 | Outer membrane protein OmpA | *ompA* | 38.396 | 5.00E-15 | | 39% | |
| Down | 1 | 3101 | Outer membrane protein W, OmpW | *ompW* | 20.097 | 3.00E-15 | | 54% | |
| Down | 1 | 3405 | Outer membrane protein OmpA | *ompA* | 38.396 | 5.00E-15 | | 39% | |
| Down | 1 | 3511 | Elongation factor Tu | *tufB* | 43.15 | 7.90E-16 | | 61% | |
| Down | 1 | 3514 | Elongation factor Tu | *tufB* | 43.15 | 7.90E-16 | | 61% | |
| Down | 1 | 5423 | TonB-linked outer membrane protein, SusC/RagA family | *fur* | 120.358 | 6.00E-06 | | 17.8 | |
| Down | 1 | 6303 | Bifunctional protein FolD | *folD* | 29.911 | 2.00E-10 | | 44% | |
| Down | 2 | 3303 | Adenylate kinase | *adk* | 24.178 | 2.50E-18 | | 70% | |
| Down | 2 | 5416 | Fructose-1,6-bisphosphate aldolase, class II | *fda* | 37.341 | 6.60E-05 | | 23% | |
| Down | 2 | 5520 | Cysteine desulfurase | [*iscS*](https://www.uniprot.org/uniprot/B0VD51) | 44.996 | 8.60E-08 | | 28% | |
| Down | 2 | 5811 | ND | ND | ND | ND | | ND | |
| Down | 2 | 5815 | Isocitrate dehydrogenase, NADP-dependent | *idh* | 82.626 | 1.06E-08 | | 23.1 | |
| Down | 2 | 6406 | Uncharacterized protein | ND | 10.964 | 1.99E-06 | | 45.5 | |
| Down | 2 | 6521 | Imidazolonepropionase | *hutI* | 44.374 | 3.10E-12 | | 46% | |
| Down | 2 | 6609 | Uncharacterized protein | ND | ND | ND | | ND | |
| Down | 2 | 6701 | Uncharacterized protein | ND | ND | ND | | ND | |
| Down | 2 | 6710 | ND | *ND* | ND | ND | | ND | |
| Down | 2 | 6802 | Isocitrate dehydrogenase, NADP-dependent | *idh* | 82.639 | 3.10E-12 | | 26.40% | |
| Down | 2 | 7509 | Succinyldiaminopimelate transaminase | *dapC* | 43.778 | 3.10E-12 | | 21.30% | |
| Down | 2 | 7602 | Dihydrolipoyl dehydrogenase | F931_01045 | 51.012 | 7.28E-06 | | 24.30% | |
| Down | 2 | 7607 | Dihydrolipoyl dehydrogenase | F931_01045 | 51.012 | 7.28E-06 | | 32.10% | |
| Down | 4 | 1513 | Succinate--CoA ligase | *sucC* | 41.675 | 1.60E-20 | | 61% | |
| Down | 4 | 5411 | Succinate-CoA ligase, alpha subunit | *sucD* | 30.594 | 9.60E-04 | | 39.9 | |
| Down | 4 | 6215 | Hypothetical protein | ND | ND | ND | | ND | |
| Down | 4 | 6219 | Hypothetical protein | ND | ND | ND | | ND | |
| Down | 4 | 6405 | Succinate--CoA ligase [ADP-forming] subunit alpha | *sucD* | 30.594 | 4.99E-06 | | 39.9 | |
| Down | 4 | 6511 | Hypothetical protein | ND | ND | ND | | ND | |
| Down | 4 | 6707 | Urocanate hydratase | *hutU* | 61.593 | 1.60E-29 | | 46% | |
| Down | 4 | 7502 | Glutamate dehydrogenase | *gdh* | 46.165 | 1.18E-10 | | 36.60% | |
| Down | 4 | 7510 | Uncharacterized protein | ND | ND | ND | | ND | |

**Supplementary File 1i.**

| **Antibiotics (µg/ml)/**  **strain** | **Polymyxin B** | **Colistin** | **Ampicillin** | **Gentamicin** | **Spectinomycin** | **Kanamycin** | **Chloramphenicol** | **Clindamycin** | **Rifampicin** |
| --- | --- | --- | --- | --- | --- | --- | --- | --- | --- |
| Lab-WT | 2 | 1 | 150 | 8 | 128 | 1 | 512 | 32 | 12 |
| PMR^Low^ | 4 | 1 | 150 | 8 | 128 | 1 | 512 | 32 | 12 |
| PMR^High^ | 128 | 32 | 150 | 8 | 128 | 1 | 512 | 32 | 12 |
| F-1025 | 4 | 2 | >150 | >16 | >128 | >1 | >512 | >64 | 12 |
| F-1208 | 2 | >16 | >150 | >16 | >128 | >1 | >512 | >64 | >48 |
| F-1379 | 2 | 1 | >150 | >16 | >128 | >1 | >512 | >64 | 12 |
| F-1410 | 8 | 2 | >150 | >16 | >128 | >1 | >512 | >64 | 12 |
| F-1629 | 128 | >64 | >150 | >16 | >128 | >1 | >512 | >64 | >48 |

**Supplementary File 1j.**

| Strain | Description | Reference |
| --- | --- | --- |
| Lab-WT | Wild-type, laboratory-cultured *A. baumannii* ATCC 17978 | Laboratory stock |
| PMR^Low^ | Isolated from 2 μg/mL PMB containing agar plate | This study |
| PMR^High^ | Isolated from 16 μg/mL PMB containing agar plate | This study |
| Δ*pmrB^w^* (Δ*pmrB*) | *pmrB* (encoded by ATCC1_00956) mutant; insertion of pVIK112-*pmrB* into Lab-WT | This study |
| Δ*pmrB^H^* | *pmrB* (encoded by ABA17978_00959) mutant; insertion of pVIK112-*pmrB* into PMR^High^ | This study |
| Lab-WT*+* pRK415 | Complementation strain; insertion of empty vector, pRK415, into Lab-WT | This study |
| Lab-WT*+* pRK-*pmrB* | Complementation strain; insertion of empty vector, pRK415, into Lab-WT | This study |
| Δ*surE* | *surE* (encoded by ATCC1_01296) mutant; insertion of pVIK112-*surE* into Lab-WT | This study |
| Δ*lpp* | *lpp* (encoded by ATCC1_01297) mutant; insertion of pVIK112-*nlpD* into Lab-WT | This study |
| Δ*fabH* | *fabH* (encoded by ATCC1_03686) mutant; insertion of pVIK112-*fabH* into Lab-WT | This study |
| Δ*ftsL* | *ftsL* (encoded by ATCC1_00505) mutant; insertion of pVIK112-*ftsL* into Lab-WT | This study |
| Δ*udg* | *udg* (encoded by ATCC1_00328) mutant; insertion of pVIK112-*udg* into Lab-WT | This study |
| Plasmids | **Description** | **Reference** |
| pVIK112 | Km^r^, R6K, *oriV*, suicide vector, *lacZ* fusion |  |
| pRK415 | Tc^r^, *oriV*, broad-host-range vector |  |
| pCasAb-apr | Apr^r^, *oriV*, broad-host-range vector | Wang et al., 2019 |
| pSGAb-km | Km^r^*, ColE1, WH1266* | Wang et al., 2019 |
| pVIK112-*pmrB^W^* | Km^r^, internal *pmrB* fragment (encoded by ATCC1_00956) in pVIK112 | This study |
| pVIK112-*surE* | Km^r^, internal *surE* fragment (encoded by ATCC1_01296) in pVIK112 | This study |
| pVIK112-*lpp* | Km^r^, internal *nlpD* fragment (encoded by ATCC1_01297) in pVIK112 | This study |
| pVIK112-*fabH* | Km^r^, internal *fabH* fragment (encoded by ATCC1_03686) in pVIK112 | This study |
| pVIK112-*ftsL* | Km^r^, internal *ftsL* fragment (encoded by ATCC1_00505) in pVIK112 | This study |
| pVIK112-*udg* | Km^r^, internal *udg* fragment (encoded by ATCC1_00956) in pVIK112 | This study |
| Knockout primers | **Sequence (5′-3′)** | **Reference** |
| *pmrB*_KO_F (*KpnI*) | CCGGAATTCGAACCGTTCTTGGTGACTGCAC | This study |
| *pmrB*_KO_R (*EcoRI*) | TCCCCCGGGCGCCAAATGAACAACACGCA | This study |
| *surE*_KO_F (*KpnI*) | CCGGAATTCTAATGGCACACCAGCCGATT | This study |
| *surE*_KO_R (*EcoRI*) | TCCCCCGGGACGCTGTGGATCAGTTACCG | This study |
| *nlpD*_KO_F (*KpnI*) | CCGGAATTCGAACCGTTCTTGGTGACTGCAC | This study |
| *nlpD*_KO_R (*EcoRI*) | TCCCCCGGGCGCCAAATGAACAACACGCA | This study |
| *fabH*_KO_F (*KpnI*) | CCGGAATTCTCTGCAACAAGCCCAGATGA | This study |
| *fabH*_KO_R (*EcoRI*) | TCCCCCGGGCCTGAGCATCTTCACTGCCA | This study |
| *ftsL*_KO_F (*KpnI*) | CCGGAATTCGAACCGTTCTTGGTGACTGCAC | This study |
| *ftsL*_KO_R (*EcoRI*) | TCCCCCGGGCGCCAAATGAACAACACGCA | This study |
| *udg*_KO_F (*KpnI*) | CCGGAATTCGAACCGTTCTTGGTGACTGCAC | This study |
| *udg*_KO_R (*EcoRI*) | TCCCCCGGGCGCCAAATGAACAACACGCA | This study |
| qRT-PCR primers | **Sequence (5′-3′)** | **Reference** |
| *mlaD*_qRT PCR_F | ACGAGCAGGTTCTGCATTGA | This study |
| *mlaD*_qRT PCR_R | ATACCAGCCCAAAGAGGTGG | This study |
| *lpp*_qRT PCR_F | AATGGACAACTCGCAGCCAT | This study |
| *lpp*_qRT PCR_R | CCCGCTTTGGCAATACGAAG | This study |
| Wang, Yu., Wang, Z., Chen,Y., Hua ,X., Yu, Y. and Ji, Q. 2019. A highly efficient CRISP Cas9-based genome engineering platform in *Acinetobacter baumannii* to understand the H_2_O_2_-sensing mechanism of OxyR. *Cell Chem Biol.* 26,1732-1742. | | |

**Supplementary File 1k.**

| **Product size (bp)** | **Sequence primers** | **Sequence (5′-3′)** | **Reference** |
| --- | --- | --- | --- |
| *pmrB*  (1,484) | *pmrB*_F | GAGGACTGGGCTACCGTTTG | This study |
|  | *pmrB*_R | GCTGGGTCGTTTGGGCAATA | This study |
|  | *pmrB_*Intermidiate | ACGCGATTCCGAAGAACTCA | This study |
| *dgcB*  (189) | *dgcB*_F | AGATGCACGTCTCTTAGCCG | This study |
|  | *dgcB* _R | ACATTTCGTCCCCGAGCTTT | This study |
| *ftsK*  (303) | *ftsK*_F | GCAGCCGTTTATTTGCGAGA | This study |
|  | *ftsK*_R | GTGGTAAACTAGGCCCGACC | This study |
| *surE*  (582) | *surE­*_F | GCGTTAAAACCACTAGGCCG | This study |
|  | *surE­*_R | ACGCTGTGGATCAGTTACCG | This study |
| *rimJ*  (136) | *rimJ* _F | ATGCATGGGGTGTGGGTTAT | This study |
|  | *rimJ* _R | ACTTGTTGTGACCTCACGTT | This study |
| *mlaC*  (579) | *mlaC*_F | GTGAGCTGGCCGTAGGTATC | This study |
|  | *mlaC*_R | CTGCGCCACCACCAGTAATA | This study |
| *fimT*  (464) | *fimT*_F | AGTTGAGCTCACTATAACACTCGT | This study |
|  | *fimT*_R | GCACATGGCCCATTTTGCTA | This study |
| *ftsL*  (319) | *ftsL*_F | AGCAGTGATGAAATCGAAACCAC | This study |
|  | *ftsL*_R | TTTGCTCTGAGGTCATCGGT | This study |
| *udg*  (493) | *udg*_F | TGAAATGGGAGCCGATTACCC | This study |
|  | *udg*_R | GGAGCTACTGTAACCGCAGG | This study |
| *fabH*  (481) | *fabH*_F | TTAAAAGCAGGCCGCTCTGG | This study |
|  | *fabH*_R | CTGCCAGCATGAGATCCTGT | This study |
| *xerD*  (335) | *xerD*_F | CGTATTCCGAGCCCTGTTCA | This study |
|  | *xerD*_R | GCTCGGCCAATTTTTGGTGA | This study |
| *feoB*  (534) | *feoB*_F | ACACAAGTCGGTTTAGCCTCA | This study |
|  | *feoB*_R | TACCAAGGCGATGCGCTAAT | This study |
| *relA*  (562) | *relA*_F | ACACAAGTCGGTTTAGCCTCA | This study |
|  | *relA*_R | TACCAAGGCGATGCGCTAAT | This study |
| Citrate-proton symporter  (221) | Citrate-proton symporter_F | TGGATGTCGTTAATAACCGTAGG | This study |
|  | Citrate-proton symporter_R | ACCCAAGAAAATAGCGCCGA | This study |
| *rsmI*  (402) | *rsmI*__F | GGCCTACCAAGTGACCGTTT | This study |
|  | *rsmI*_R | AGCCAGTTGAGATGCTGCTT | This study |
| *folA*  (423) | *folA*_F | TGTCGAAGGTCCAAATGGGG | This study |
|  | *folA* _R | CTACCAATCAGCACAGGCGA | This study |
| *guaA*  (439) | *guaA_*F | AACACAACAAACGCCTTCCC | This study |
|  | *guaA* _R | TGTCGCCATGCCAGTGTAAA | This study |
