# Supplementary material for: A novel decoy strategy for polymyxin resistance in Acinetobacter baumannii
Source: eLife. 2021 Jun 28;10:e66988. doi: 10.7554/eLife.66988 (PMC8324293; doi:10.7554/eLife.66988)
Supplement: Transparent reporting form [file elife-66988-transrepform.docx]

***eLife’s* transparent reporting form**

We encourage authors to provide detailed information *within their submission* to facilitate the interpretation and replication of experiments. Authors can upload supporting documentation to indicate the use of appropriate reporting guidelines for health-related research (see [EQUATOR Network](http://www.equator-network.org/%20)), life science research (see the [BioSharing Information Resource](https://biosharing.org/)), or the [ARRIVE guidelines](http://www.plosbiology.org/article/info:doi/10.1371/journal.pbio.1000412) for reporting work involving animal research. Where applicable, authors should refer to any relevant reporting standards documents in this form.

If you have any questions, please consult our Journal Policies and/or contact us: [editorial@elifesciences.org](mailto:editorial@elifesciences.org).

**Sample-size estimation**

- You should state whether an appropriate sample size was computed when the study was being designed
- You should state the statistical method of sample size computation and any required assumptions
- If no explicit power analysis was used, you should describe how you decided what sample (replicate) size (number) to use

Please outline where this information can be found within the submission (e.g., sections or figure legends), or explain why this information doesn’t apply to your submission:

This information is contained in the Methods subsection and used student’s t-test as a statistical method. Resistant strains to the presence of up to 16 μg/ml polymyxin B (PMB) were obtained by using a continuous plate culture method. To ensure the representativeness of these strains, 10 isolates were obtained from Lab-WT, PMR^Low^, and PMR^High^, respectively, and the MIC test was examined (Figure 1).

**Replicates**

- You should report how often each experiment was performed
- You should include a definition of biological versus technical replication
- The data obtained should be provided and sufficient information should be provided to indicate the number of independent biological and/or technical replicates
- If you encountered any outliers, you should describe how these were handled
- Criteria for exclusion/inclusion of data should be clearly stated
- High-throughput sequence data should be uploaded before submission, with a private link for reviewers provided (these are available from both GEO and ArrayExpress)

Please outline where this information can be found within the submission (e.g., sections or figure legends), or explain why this information doesn’t apply to your submission:

The independent biological replicates are indicated with the error bars in the figure legend (Figures 1, 4, 6 and 7) and in the ‘Material and Methods’ section (*Galleria mellonella* infection model). More than triplicate experiments were performed in MIC test (Figure 1A) and OMV quantification (Figure 1C, Figure 4A-F). Additional statement concerning reproducibility of the results is provided in the “Materials and Methods” section.

**Statistical reporting**

- Statistical analysis methods should be described and justified
- Raw data should be presented in figures whenever informative to do so (typically when N per group is less than 10)
- For each experiment, you should identify the statistical tests used, exact values of N, definitions of center, methods of multiple test correction, and dispersion and precision measures (e.g., mean, median, SD, SEM, confidence intervals; and, for the major substantive results, a measure of effect size (e.g., Pearson's r, Cohen's d)
- Report exact p-values wherever possible alongside the summary statistics and 95% confidence intervals. These should be reported for all key questions and not only when the p-value is less than 0.05.f

Please outline where this information can be found within the submission (e.g., sections or figure legends), or explain why this information doesn’t apply to your submission:

Each statistical test, values of N, and p-values are provided in the appropriate figure legends (Figure 1C and Figure 4A-F) and Materials and Methods. For each experiment * means p-value< 0.05 indicated in the figure legend.

(For large datasets, or papers with a very large number of statistical tests, you may upload a single table file with tests, Ns, etc., with reference to sections in the manuscript.)

**Group allocation**

- Indicate how samples were allocated into experimental groups (in the case of clinical studies, please specify allocation to treatment method); if randomization was used, please also state if restricted randomization was applied
- Indicate if masking was used during group allocation, data collection and/or data analysis

Please outline where this information can be found within the submission (e.g., sections or figure legends), or explain why this information doesn’t apply to your submission:

Experimentally evolved PMB-resistant isolates were grouped according to MIC (Figure 1A). This is indicated in the Materials and Methods.

**Additional data files (“source data”)**

- We encourage you to upload relevant additional data files, such as numerical data that are represented as a graph in a figure, or as a summary table
- Where provided, these should be in the most useful format, and they can be uploaded as “Source data” files linked to a main figure or table
- Include model definition files including the full list of parameters used
- Include code used for data analysis (e.g., R, MatLab)
- Avoid stating that data files are “available upon request”

Please indicate the figures or tables for which source data files have been provided:

Whole genome sequencing (WGS) was performed on three selective *A. baumannii* isolates. The genome sequences were downloaded from EzGenome (http://ezgenome.ezbiocloud.net) and were analyzed using the CLgenomics program (ChunLab, South Korea). Sequence reads for the resistant strains are accessible from the NCBI sequence read archives under accession no. PRJNA530195 (Lab-WT), PRJNA530197 (PMR^Low^), and PRJNA530202 (PMR^High^).

The bacterial community data have been deposited in NCBI under Sequence Read Archive (SRA) accession number SRX9819399 PRJNA689940, SRX9819397 PRJNA689944 and SRX9819398 PRJNA689944.
